# Incidence of colorectal cancer in Eritrea: Data from the National Health Laboratory, 2011-2017

**DOI:** 10.1371/journal.pone.0224045

**Published:** 2019-11-13

**Authors:** Lidia Biniam Medhin, Oliver Okoth Achila, Adiam Tesfamariam Abrham, Biniam Efrem, Kibrom Hailu, Daniel Mebrahtu Abraha, Luwam Gilazghi, Alay Meresie, Salih Mohammed Said

**Affiliations:** 1 Pathology, National Health Laboratory, Asmara, Eritrea; 2 Clinical Laboratory Sciences, Orotta School of Medicine and Health Sciences, Asmara, Eritrea; 3 Hematology, National Health Laboratory, Asmara, Eritrea; 4 Immunoserology, National Health Laboratory, Asmara, Eritrea; 5 Clinical Chemistry, National Health Laboratory, Asmara, Eritrea; 6 Microbiology, National Health Laboratory, Asmara, Eritrea; University of New Mexico, UNITED STATES

## Abstract

**Introduction:**

Recent studies have reported a significant increase in the prevalence of colorectal cancer (CRC) in Sub-Saharan Africa (SSA). Further, several studies employing disparate modelling algorithms have projected a significant rise in the frequency of CRC cases in the region. However, lack of good quality data on multiple themes related to CRC including incidence, among others, continues to be a problem in the region. Therefore, this study was designed to collect data on the incidence of CRC in Eritrea.

**Methods:**

We conducted a retrospective analysis using data captured between 2011 and 2017 at the National Health Laboratory (NHL) in Asmara, Eritrea.

**Results:**

241 colorectal cases were identified in the Eritrean National Health Laboratory (NHL) database between 2011 and 2017. In the final analysis we included 94 patients confirmed cases giving an average of 18.8 patients per annum. The average age ± Standard deviation (SD) was 57.62 ± 17.14 with a male: female ratio of 58/36 (1: 1.61). The minimum and maximum age of the patients was 19 and 90 years, respectively. The rectum to colon ratio was 47/47 (1:1). The proportion of patients < 50 years in this cohort was significant. The age-standardized incidence rate (ASIR) in the study period was between 0.97 per 100 000 to 2.21 per 100 000. Similarly, the cumulative ASIR was 9.97 per 100 000. Analysis of trends did not reveal shifts over the study period (P<0.05). However, a strong correlation between incidence and age was established.

**Conclusion:**

This study suggests that the incidence of CRC in Eritrea is relatively low. A significant number of patients were less than 50 years of age. Even then it’s our opinion that this study may underestimate the incidence of CRC.

## Introduction

According to a recent Global Burden of Cancer Study (GLOBOCAN) estimate, colorectal cancer (CRC) is the 3^rd^ most commonly diagnosed gastrointestinal malignancy worldwide [1, 2]. Reports indicate that the highest pooled incidence rates for CRC are in Western countries with Low and Medium income Countries (LMIC) reporting relatively low rates. For instance, the crude incidence rate in the US and United Kingdom (UK) are 25/100,000 and 30.2/100 000, respectively. In contrast, a recent meta-analysis reported that the crude incidence of CRC in SSA is 4.04/100,000 (4.38/100,000 for men and 3.69/100,000 for women) [3]. In particular, wide regional variation in the geographical distribution of CRC has been reported in the continent with low crude incidence rates in West Africa (Men: 4.5/100,000 and Women: 3.8/100,000) and East Africa (6.5/100,000) [1]. Similarly, higher incidence rates have been reported in North Africa (7.1/100,000) and Southern Africa (10.9/100,000) [1]. Altogether, the incidence of CRC in SSA appears to correlate strongly with a country’s level of socio-economic development [1]. To illustrate, Mauritius and South Africa (SA), the countries with some of the highest Human Development Index (HDI) in the region; have an incidence of 18.6/100,000 and 11.9/100,000, respectively [1, 4]. Altogether, the data confirms the observation that the variations in incidence and prevalence of CRC correlates strongly with socioeconomic levels.

Although the incidence of CRC in LMIC in SSA is comparatively low, the region has a higher CRC–associated mortality and morbidity. For instance, a past report indicated that of the 600, 000 deaths associated with CRC in 2008, 70% occurred in LMIC [2, 5]. In the meantime, the prevalence, incidence and resultant relevant costs such as disability-adjusted life years (DALY) due to premature death from CRC or from individuals living with CRC are projected to increase [4]. The increasing incidence and prevalence of CRC is part of the on-going epidemiological transition in SSA characterized by a reducing burden of infectious diseases coupled with a rising burden of non-communicable diseases (NCD) [2, 6]. In turn, this outcome has been linked to multiple factors including increasing longevity, rapid societal and economic changes and the presence of associated risk factors [1, 7]. These risks include alcohol consumption, hereditary factors such as familial adenomatous polyposis (FAP) and hereditary non-polyposis colorectal cancer (HNPCC), diabetes mellitus, chronic inflammatory diseases of the colon (ulcerative colitis or Crohn’s colitis, among others), poor nutrition (low consumption of fruits and vegetables, high consumption of red processed meat or high—glycemic load carbohydrates), physical inactivity, prior ovarian/endometrial cancer before age 50 and smoking, among others [2, 8, 9]. The interactions and relative contribution of each of these factors to increased risk of CRC are complex and, at times, difficult to unravel. Altogether, a substantial amount of evidence has demonstrated that the epidemiology of CRC is shifting towards increased incidence and prevalence in individuals <50 years of age [1, 4]. The earlier age at onset, apart from being more aggressive disease, has also been associated with poor prognosis.

Considering the relatively high burden of infectious diseases in SSA, the increasing burden of CRC; a chronic condition requiring long—term medical expenditures, can only exacerbate the prevailing vicious cycle of health and poverty. The social and economic impact on individuals, families, community and/or countries or regions will therefore be substantial. A recent report from a group of experts noted that in the 21^st^ century, cancer associated mortality will rank as the single most important barrier to increasing life expectancy worldwide [1]. Although, researchers generally agree that the burden of CRC in SSA will increase, the true incidence, prevalence and burden of CRC remain uncertain [2, 3]. This uncertainty has largely been attributed to the dearth of high-quality cancer registries and the limited number of studies [1, 4, 10]. Additional concern includes the possibility that data from existing registries is often suboptimal/partial or conflicting, hence, incomprehensive [4, 10]. Corroborating this conclusion, trace-back of cancer deaths to obtain the corresponding hospital records has demonstrated that quality of diagnostic information on cancer cases is generally poor [10]. Limited diagnostic infrastructure and late presentation of cases to health care providers also undermine the validity of CRC data from the region. In Eritrea, data on the incidence of CRC is lacking in international literature. Given the paucity of data on CRC and projections of an increase in SSA, understanding the incidence of CRC in Eritrea is imperative. Therefore, this study was designed to collect data on the incidence of CRC in the country.

## Method and materials

### Study design and setting

We conducted a retrospective analysis using data captured between 2011–2017 at the National Health Laboratory (NHL) in Asmara, Eritrea. NHL serves as a referral facility for all hospitals in Eritrea and has the only pathology laboratory in the country. Therefore, all cancer specimens in the country are processed at the facility. In this regard, the NHL database (Polytech 8.37.C) captures relevant data on all malignancies countrywide. Between 2011–2017, 9 403 cancer cases were recorded among these 241 were Colon (C18) and Rectum (C20) cases. The information captured in the system includes demographic characteristics (sex and age), histopathology (morphology), primary site (topography), behavior, date of diagnosis, differentiation status and stage (number staging), among others. Tumor classification is based on International Classification of Diseases (ICD) for Oncology, 3^rd^ ed. (ICD-10 and ICD-O) [11]. All the cases were also confirmed by a resident pathologist or pathologists in specific reference institutions.

### Completeness and data quality

In evaluating cancer incidence, ensuring that incident cases in the population are included is critical. In this study, we evaluated completeness using a number of strategies including stability of incidence rates over time. The use of mortality-to-incidence ratios was not possible due to lack of data on mortalities.

### Data analysis

Data extracted from NHL Polytech data base (Polytech 8.37.C) was cross-checked against a hard copy maintained at the facility. Duplicate entries were eliminated based on demographic data and patient phone number. Chi-squire (χ^2^) test was used to evaluate differences between proportions. A one-sided P value > 0.05 was considered significant. Further, the crude average annual incidence rate (CIR), was calculated by dividing the total number of cases by the corresponding population at risk, expressed per 100 000 person-years. The ASIR was calculated by the direct method, using 10-year age bands (≤ 40–49, 50–59, 60–69 and ≥ 70). ASIR conversions were based on world population net 2014. Annual trends in CRC were evaluated by the annual percentage change (APC) over time period as: [exp (β) -1] × 100). The regression coefficient (β) was estimated from a linear regression between logarithmic-transformed age-standardized cancer rates per calendar year [12]. Data was entered in Microsoft excel 2010 and analyzed using IBM, SPSS software version 20.

### Ethical approval and consent

The study was approved by the Health Research Ethics and Protocol Review Committee of the Eritrean Ministry of Health (MOH). Consent to participate was not obtained from the patients. This waiver was premised on the consideration that the study was based on anonymized patient’s records.

## Results

**Fig 1:**
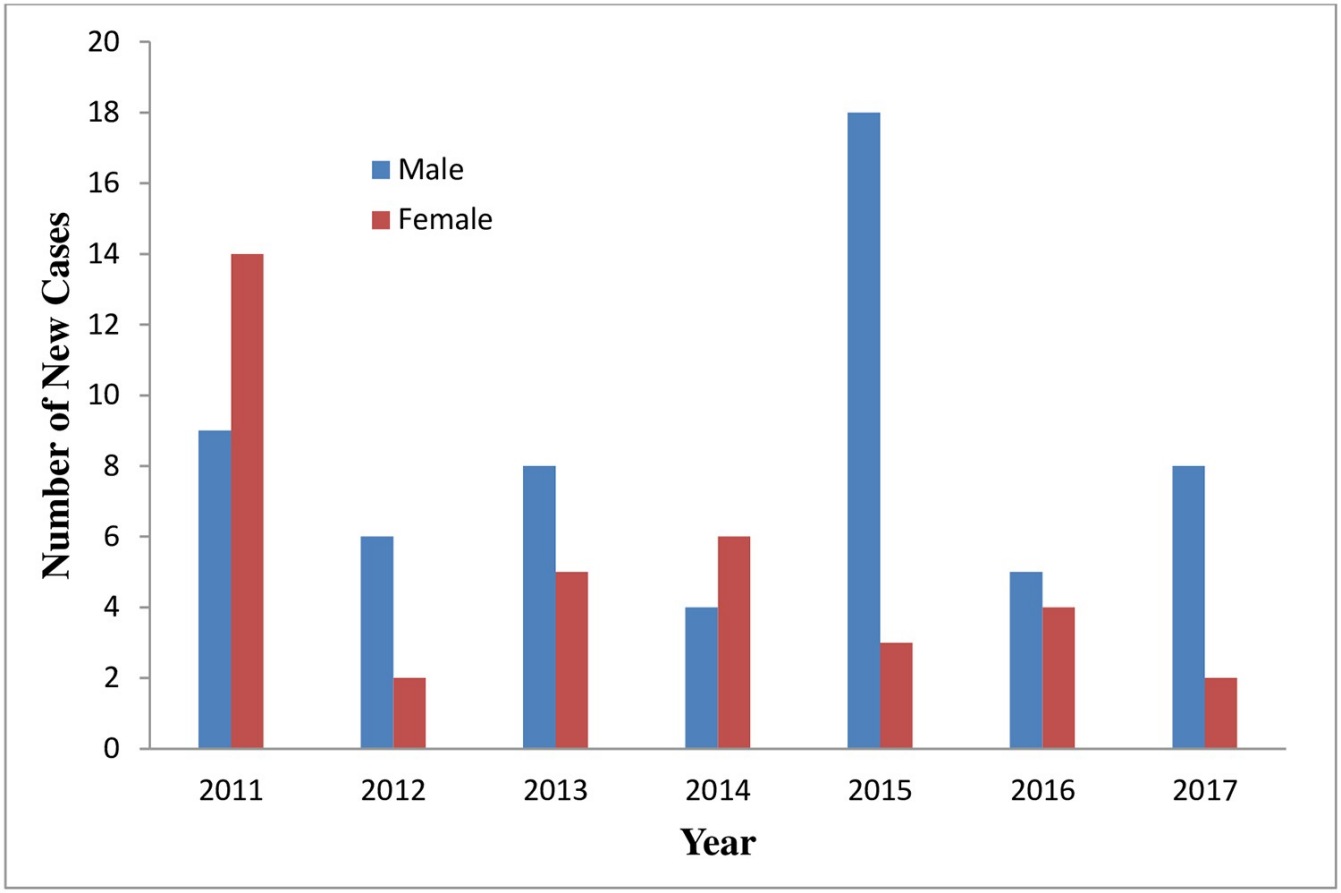
Annualised distribution of new cancer cases.

We studied 94 confirmed cases of CRC over the study period (2011–2017). The average age ± standard deviation (SD) was 57.62 ± 17.14 with a male: female ratio of 58/36 (1: 1.61). The minimum and maximum age of the patients was 19 and 90 years. The annualised distribution of cancer cases is shown in Fig 1.

Information on tumour behaviour, morphology, grade and stage was abstracted from the database. In terms of behavior, 94 (39%) of the patients had malignant tumour and 147 (61%) had benign tumour. Disaggregation of malignant tumor in terms of morphology showed that 84 (89.4%) of the tumours were adenocarcinomas, 9 (9.6%) were squamous cell carcinomas, and 1 (1.1%) were anaplastic carcinomas. Number staging system (Stages 1 and 2 as early colorectal cancer and stages 3 and 4 as progressive colorectal cancer) was used to stage the tumours. The proportions of tumour in the different stages were as follows: 13 (13.93%) of the tumours were in stage 1; 23 (24.5%) were in stage 2; 48(51.1%) were in stage 3 and 10 (10.6%) were in stage 4. Information on cancer grades are shown in Table 1. Altogether, we have to note that data on tumour grades was only available for 50% of the cases. Analysis of the distribution of the different grades and stages of cancers across the disparate age groups did not show any significant difference.

**Table 1:** Tumour characteristic.

Tumour characteristic	Patient No%
Types of Tumors	
Malignant	94 (39)
Benign	147 (61)
Morphology	
Adenocarcinoma	84 (89.4)
Squamous Carcinoma	9 (9.6)
Anaplastic Carcinoma	1 (1.1)
Grading	
Grade1(Well-differentiated)	23 (48.9)
Grade 2 (Moderately differentiated)	21 (44.7)
Grade 3 (Poorly differentiated	3 (6.38)
Stage (Number Staging)	
Stage 1	13 (13.83)
Stage 2	23 (24.5)
Stage 3	48 (51.1)
Stage 4	10 (10.6)

**Fig 2:**
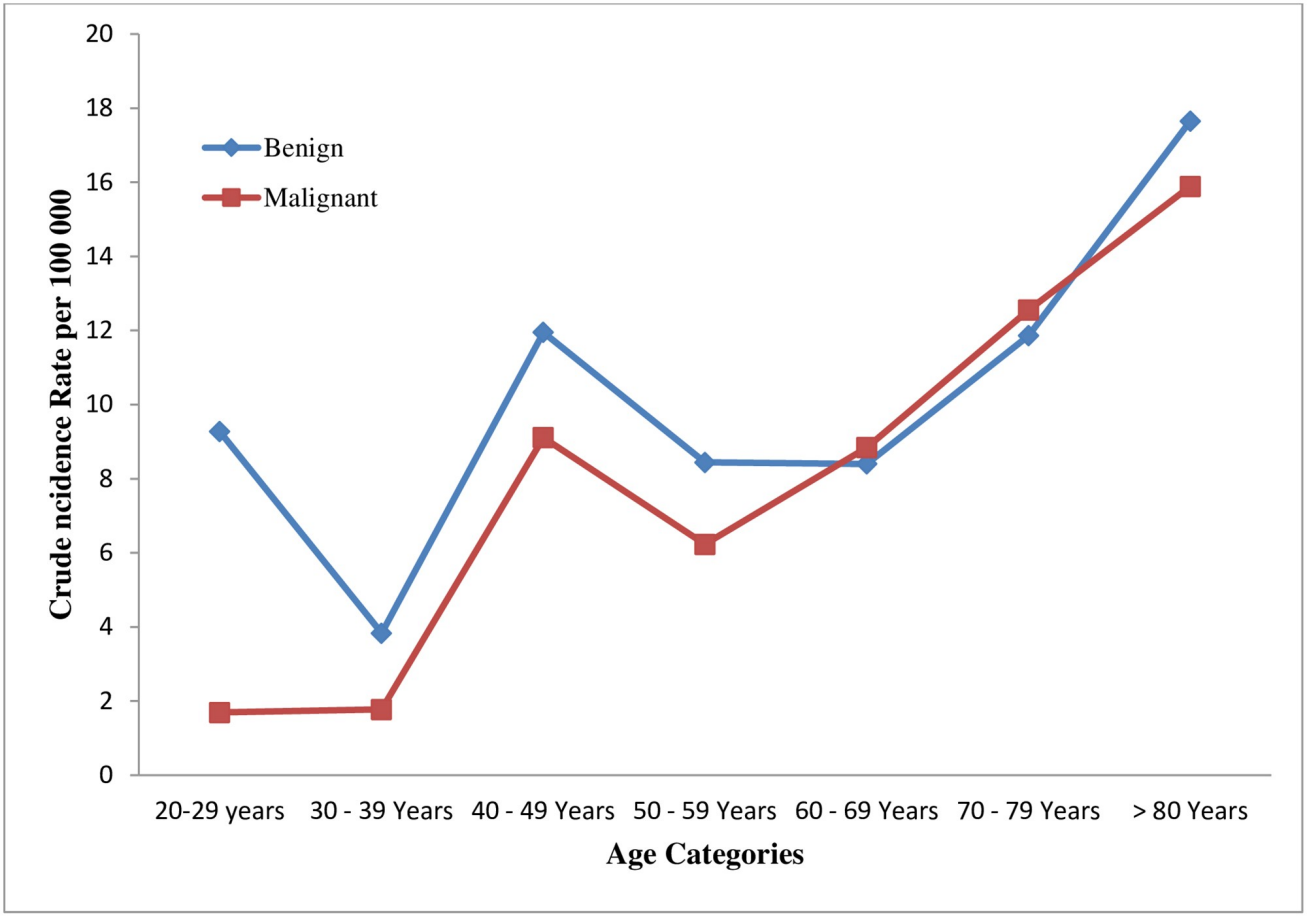
Crude incidence rate of colorectal cancers across different age categories.

The proportion of patients < 50 years in this cohort was sizable. The data showed that (8/94) 8.5% of the patients were < 29 years (male: female ratio was 6:2 (3:1)); 6/94 (6.38%) were in the 30–39 year age range (male: female ratio was 3:3 (1:1)) and patients in the 49–50 years age range were 16/94 (17.02%) (Male: female ratio was 10:6 (1.67). Most of the colon cancer cases were in the 60–69 age groups. The highest cumulative CIR was observed in patients in the >80 years age band(Fig 2).

Disaggregation by sex showed that males were more likely to present with CRC compared to women. In males, the highest CIR and ASIR was in individuals > 80 years of age. In contrast, the highest rate in females was observed in the 60–69 years age band. Overall, the highest ASIR was observed in patients in the 70–79 years age band. The cumulative ASIR over the study period for males and females were 15.75 and 6.23 per 100 000, respectively. Similarly, the cumulative ASIR for both males and females in the study period was 9.92 per 100 000 (Table 2).

**Table 2:** Seven year age standardised incidence rates and crude incidence rates of colorectal cancer by age groups for both sexes (per 100 000 population).

Year Bands	Male		Females		Both	
	CIR	ASIR	CIR	ASIR	CIR	ASR
**<40 Years**	2.08	0.63	1.14	0.32	1.61	0.47
**40–49 Years**	7.57	2.02	4.27	1.17	5.87	1.59
**50–59 Years**	6.42	1.31	8.53	1.69	7.51	1.51
**60–69 Years**	15.39	2.06	12.76	1.75	13.95	1.89
**70–79 Years**	63.65	4.30	11.54	0.90	31.77	2.31
**>80 Years**	226.50	5.43	11.39	0.40	73.10	2.15
**2011–2017**		15.75		6.23		9.97

**ASIR**: Age standardised Incidence Rate; **CIR**: Crude incidence rate

### Rates per 100 000 for colon and rectal cancers

There were 47 colon and 47 rectal cancer cases giving a rectum to colon ratio of 47/47 (1:1). Trend analysis did not demonstrate significant changes (P value > 0.05) in the magnitude of colonic or rectal tumours during the study period. Additional information on the distribution of colonic and rectal cancers is shown in Table 3.

**Table 3:** Annualised crude incidence rate per 100,000.

Years	Colon	CIR	ASIR	Rectum	CIR	ASIR	Both	CIR	ASIR
**2011**	11	0.70	1.19	12	0.76	1.27	23	1.45	2.21
**2012**	4	0.25	0.42	4	0.25	0.55	8	0.51	0.97
**2013**	5	0.32	0.65	8	0.51	0.67	13	0.82	1.32
**2014**	5	0.32	0.48	5	0.32	0.63	10	0.63	1.12
**2015**	9	0.57	1.00	12	0.76	1.14	21	1.33	2.13
**2016**	6	0.38	0.56	3	0.19	0.24	9	0.57	0.93
**2017**	7	0.44	0.76	3	0.19	0.5	10	0.63	1.30
**2011–2017**	47		5.06	47		5.0	94		9.98

**ASIR:** Age standardised incidence rate; **CIR**: Crude incidence rate

Annualised ASIR across different age bands for the study duration are shown in Table 4. Analysis of trends did not exhibit year to year changes in the magnitude of CRC.

**Table 4:** Annualised age standardised rates for each age group per year.

Colorectal Cancer	Age Group (Years)						
	<40	40–49	50–59	60–69	70–79	> 80	All
**Overall**							
**2011**	0.13	0.4	0.36	0.57	0.51	0.24	2.21
**2012**	-	0.20	0.18	0.09	0.26	0.24	0.97
**2013**	0.07	0.20	0.27	0.28	0.26	0.24	1.32
**2014**	0.03	0.40	-	0.19	0.26	0.24	1.12
**2015**	0.13	0.20	0.53	0.28	0.51	0.48	2.13
**2016**	0.03	0.1	0.18	0.38	-	0.24	0.93
**2017**	0.03	0.1	0.09	0.09	0.51	0.48	1.30

## Discussion

CRC incidence and associated mortality are growing in SSA. This phenomenon has been traced to a complex interrelationship between multiple factors associated with the rapidly evolving socioeconomic transition in the region [1]. In this regard, increasing incidence of CRC in SSA is an important marker of the public health challenges associated with the on-going epidemiological transition in SSA. Without doubt, it serves to underscore the importance of continued surveillance of CRC in the region. Against this backdrop, this article provides a status report on the incidence of CRC in Eritrea in 2011–2017. The data indicates that the CIR ranged between 0.97 per 100 000 to 2.21 per 100 000. The low incidence rate reported in this study parallels findings from specific countries in SSA (Gambia and Mozambique (both 1.5 per 100 000)) [2]. Our results are also consistent with the findings by previous investigators in the country [13] and with the assertion that colon cancers are rare in native African (NA) population’s [1]. Instructively, the estimates are lower compared to GLOBOCAN averages for the region [1]. Importantly, we have to note that GLOBOCAN reports on Eritrea are modelled using data from cancer registries from neighbouring countries (Ethiopia in particular). The ratio of CRC between sexes also reflected established patterns indicating a high prevalence in males compared to women 58/36 (1: 1.61). Underscoring this disparity, a recent GLOBOCAN report noted that the age-standardized incidence rate is about 1.4 times higher in men than in women. In contrast, Graham *et al*. estimated that the male: female ratio of CRC in SSA is 1.17 [4]. Additionally, analysis of trends did not reveal shifts over the study period (p<0.05). This result might be due, in part, to limited statistical power.

A salient consideration that should inform the evaluation of the representativeness of our estimates is that it is largely based on diagnostic and not on screening evaluations. Similar to other countries in SSA, Eritrea lacks a system-wide, organised approach to screening—programmatic and opportunistic screenings are marginal. The complete absence or marginal use of screening tools such as faecal immunochemistry testing (FIT); faecal occult blood testing (FOBT); fecal DNA, capsule endoscopy, colonoscopy, flexible sigmoidoscopy and CT colonography is particularly noteworthy. Lack of access to medical services, low level of awareness, dearth of specialists (gastroenterologists, surgeons, oncologists, and radiation oncologists) and limited utilization of existing, albeit, limited services are important barriers.

Late presentation of CRC cases also appears to be a common feature in Eritrea. A fact reflected in the low ratio between benign and malignant cases 147/94 (1.56) reported in this study and the high proportion of patients (61.7%) with advanced disease (Stage 2 and above). For this reason, it’s our opinion that this result may underestimate the incidence of CRC in the country. Reflecting on this phenomenon, several commentators have noted that the greatest concern related to cancer incidence reports from SSA relates to the systematic under-reporting of cases due to lack of high quality population-based cancer registries (PBCRs) [1]. The low incidence may also be attributed to the low level of screening in the continent.

Overall, this study highlights the need for screening services in the country. The introduction of low cost screening modalities such as guaiac faecal occult blood tests (gFOBT) and faecal immunochemical test (FIT) should be explored. The fact that such programs should target the elderly population in the country is suggested by the relatively high ASR observed in patients > 50 years (Table 1). Multiple reports indicate that systematic application of these techniques either within the framework of a programmatic or opportunistic screening program may enhance detection of undiagnosed CRC [1, 4]. Subsequent removal of pre-neoplastic lesions (polypectomies) may ultimately lower the incidence of CRC in these populations. Regrettably, cancer screening services rank low in the hierarchy of public health priorities in most countries in SSA.

Another important finding in our study is the significant number of patients < 50 years (the breakpoint employed by the Amsterdam criteria for identifying patients with increased odds of developing Lynch Syndrome (LS)—a highly penetrant hereditary cancer syndrome) [14] presenting with CRC. The changing pattern of CRC in SSA including the increasing number of cases in individuals < 50 years has been reported [14,15,16]. This finding may have a dual interpretation. First, it may be a consequence of the prevailing socio-economic transition in the Country. Much has been written about the link between global variation in CRC and westernisation of lifestyles and diets [4, 14]. In line with this thinking, the increasing prevalence of CRC in SSA including the rising incidence among individual’s ≤ 50 years may be an outcome of the dynamics associated with the changing lifestyles [12, 17]. Second, the data suggests the possibility that a proportion of CRC cases in Eritrea are inherited. Reports indicate that up to one-third of the CRC risk may be attributable to hereditary factors and approximately 3~5% of all CRCs are due to hereditary syndromes [14, 18, 19]. Hereditary non-polyposis colorectal cancer (HNPCC); Lynch syndrome (mutations in DNA mismatch repair (MMR) genes, MLH1, MSH2, PMS1, PMS2, MSH6 and EPCAM) [20,21,22,23,24]; familial adenomatous polyposis coli (FAP) (specific germline mutations in APC genes–constitutive activation of *Wnt* pathway), Cowden syndrome, among others have been implicated in the latter [14, 20, 25, 26]. However, the connection between family history or known syndromes and *de novo* occurrence of CRC in SSA is generally under-investigated. Emphasising this fact, a study involving a small cohort of CRC patients in Nigeria and South Africa (SA) suggested that HNPCC may be more frequent than hitherto reported [24, 27]. Recognizing the controversy associated with the contribution of hereditary factors to CRC cases in SSA, future research should focus on molecular profiles of CRC in young patients in Eritrea. Investigating the pharmacogenomics profiles of CRC in Eritrea may also inform therapeutic option.

### Strength and limitation

In this study, we have attempted to provide a snapshot of the incidence and trend of CRC using the most reliable data source in Eritrea. In a general sense, the data from NHL captures information from all CRC patients in all the national and regional referal hospitals in the country. Given that a majority of these cases were referred by doctors based on the presentation of specific symptoms, the data may underestimate incidence of CRC in the country. Another underlying assumption in this study is that all specimens from CRC cases in Eritrea are inevitably referred to the facility. This may not be true especially for patients from remote parts of the country. However, it’s our opinion that the data from NHL is superior to the alternative option of data collection through hospital–based registries. Another weakness stems from the lack of granularity in our analysis. Importantly, a separate assessment of variations in proximal (right-sided) cancers of the descending sigmoid colon and distal (left-sided) cancers of the rectosigmoid junction among others was not attempted. The sourcing of population denominators used in this study from population pyramid net of 2014 may also be limiting.

## Conclusions

This study suggests that the crude incidence rate of CRC in Eritrea is relatively low. Moreover, analysis of trends did not reveal shifts over the study period (2011–2017). However, it’s our opinion that this study may underestimate the incidence of CRC in Eritrea. Even then, the study provides critical baseline information that may help in creating research platforms for risks assessment or identification of putative targets for intervention. Further, a majority of the patients had advanced disease. Altogether, the study suggests that research on cases involving patients who are <50 years is vital. The possibility of establishing aggressive screening programs even for patients < 50 years should be considered. The overwhelming need to expand and integrate primary prevention (public awareness and physicians sensitisation, among others) and early detection measures(screening modalities) into existing healthcare plans and improve data collection gaps exists.

## Supporting information

S1 File The detailed information on the raw data used for the research. (SAV)

S2 File The detailed information used to create Fig 1. (XLSX)

S3 File The detailed information used to create Fig 2. (XLSX)

## Abbreviations

APC: Adenomatous polyposis coli
ASR: Age standardised rates
CRC: Colorectal cancer
CT: computer Tomography
FAP: familial adenomatous polyposis coli
FIT: Faecal immunochemistry testing
FOBT: Faecal occult blood testing
HNPCC: Hereditary non-polyposis colorectal cancer
LMIC: Low and Medium Income Countries
NHL: National Health Laboratory
PBCR: Population-based cancer registry
SSA: sub-Saharan Africa
